# Small Fluxgate Magnetometers: Development and Future Trends in Spain

**DOI:** 10.3390/s100301859

**Published:** 2010-03-09

**Authors:** David Ciudad, Marina Díaz-Michelena, Lucas Pérez, Claudio Aroca

**Affiliations:** 1 ISOM - ETSI Telecomunicación, Universidad Politécnica de Madrid (UPM), Ciudad Universitaria s/n, 28040 Madrid, Spain; E-Mail: caroca@fis.upm.es; 2 School of Physics & Astronomy, E. C. Stoner Laboratory, University of Leeds, Leeds, LS2 9JT, UK; 3 Instituto Nacional de Técnica Aeroespacial (INTA), Ctra. Torrejón-Ajalvir km 4.2, 28850 Madrid, Spain; E-Mail: diazma@inta.es; 4 Departamento Física de Materiales, Universidad Complutense de Madrid, Madrid 28040, Spain; E-Mail: lucas.perez@fis.ucm.es

**Keywords:** magnetometry, fluxgate, planar fluxgate, signal processing, double in-phase demodulation, automatic identification, automatic guidance, RFID, space applications

## Abstract

In this paper, we give an overview of the research on fluxgate magnetometers carried out in Spain. In particular we focus in the development of the planar-type instruments. We summarize the fabrication processes and signal processing developments as well as their use in complex systems and space.

## 1. Introduction

Fluxgates are directional magnetic sensors based on the non-linearity of the magnetization of soft-ferromagnetic materials. Today their sensitivity spans a wide range from 10^−10^ to 10^−4^ T. Fluxgate sensors were developed in the 30s and were rapidly used for a number of applications during the Second World War: airborne magnetic surveys, submarine and UneXploded Ordinance (UXO) detection. After the war new fluxgates were applied for geomagnetism prospection, navigation, and mapping of the Earth’s magnetic field. At the start of the space age, fluxgates began to be used as payloads of satellites for the in-orbit measurement of the Earth magnetic field, for navigation and for the measurement of interplanetary magnetic fields [1]. For a description of the principles and achieved parameters in different devices see [2,3]. At present, the efforts to improve fluxgate magnetic sensors are mainly aimed at their miniaturization. In this context, the planar fluxgate has an increasing importance since it allows a better integration of the sensor and the electronics as well as reducing the fabrication cost.

**Figure 1.**
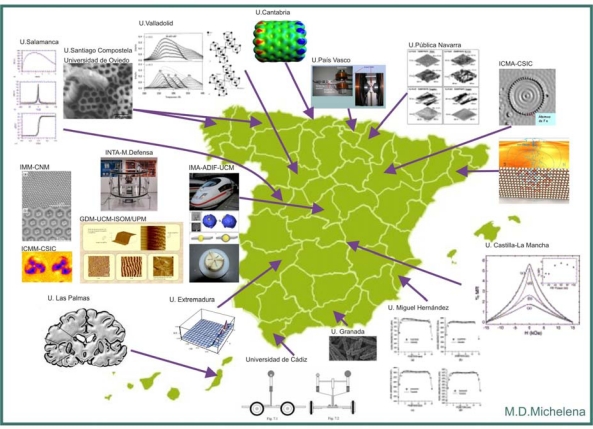
“Spanish Magnetic Map”.

In Spain, fluxgates have been used in different research projects like in geophysics [4,5], in aircrafts and in Unmanned Aerial Vehicles (UAV). While there are widespread research efforts in Spain in the area of magnetism and magnetic materials, only a few select groups have focused on fluxgate development. Figure 1 shows some of the different research groups in magnetism in Spain and their location. In the 90s some work in fluxgates was carried out by Prof. B. Hernando Grande *et al.* from the Department of Physics at the University of Oviedo [6,7]. It was coupled with a long standing collaboration with the Group of F. Primdahl and O.V. Nielsen, from the Technical University of Denmark. The collaboration started in the late 80s when Prof. B. Hernando worked at the Danish Institute [8]. The group from the University of Oviedo has also studied magnetic materials for their use in fluxgates [9–11].

The main efforts in the development of fluxgates in Spain are due to the Group of Magnetic Devices (GDM) from the Complutense (UCM) and the Polytechnic University of Madrid (UPM). Their work in fluxgates started in the mid 90s with the development of the first planar fluxgate [12], some new signal processing procedures [13], and the study of electrodeposited soft magnetic materials for their use in fluxgates cores. This research was continued with the miniaturization of the fluxgates. The GDM has also used their fluxgates and signal processing techniques in different applications.

Here we give an overview of the work on planar fluxgates in Spain, their signal processing and systems development with these magnetic sensors. Finally we describe their use in space applications at the Spanish National Institute for Aerospace Technology (INTA).

## 2. Planar Fluxgates

Reducing the size and weight of magnetic sensors is essential in military and aerospace technologies and in the microelectronics industry. Within this context, the future development of fluxgates is closely linked to the possibility of reducing their size and making them compatible with microelectronics technology. In Spain, the search for small and reliable fluxgates started in the mid 90s with the report by Vincueria *et al.*, of a fluxgate sensor based on planar technology [12]. In this section, we give an overview of the efforts made in Spain, mainly at the GDM in Madrid, to develop planar fluxgate sensors.

There are two main problems to overcome in the miniaturization of a fluxgate sensor: (1) the miniaturization of the coils and (2) the integration of the ferromagnetic core in the device. There are two basic approaches for the miniaturization of the coils. One possibility consists in using planar coils in several layers, with the magnetic core sandwiched between them. The other approach is based on the use of several fabrication steps to make spiral coils surrounding a ferromagnetic thin film that acts as a sensing core.

The first approach was used in the previously cited paper by Vincueria *et al.* The planar device described in this paper is composed of planar exciting and detecting windings with two amorphous ribbons (Metglas 2705M) acting as cores, sandwiched between the windings. The sensor performance was fairly good, showing a good linear range (with the linearity error smaller than 0.16 for magnetic field bellow 40 μT), and good sensitivity (500 V/T with a driving current of 8 mA and 1 kHz). The sensitivity is increased asymptotically to 1200 V/T when raising the frequency of the driving current. The authors suggest that it would be interesting to explore the possibility of growing the ferromagnetic core directly on the boards which support the windings, adjusting their size and placing them in a region where the excitation field is homogeneous. Following up on this idea, an attempt was made some years later to miniaturize this kind of devices sensor using a combination of UV-photolithography, sputtering and electrodeposition.

In 2003, Almazán *et al.* reported a planar inductor with attractive characteristics to be used as excitation coil for a planar fluxgate sensor [14]. The Cu coil was fabricated on a glass substrate by sputtering a Cr/Cu thin film subsequently patterned by photolithography. Electroplating was used to increase the thickness of the Cu tracks, reducing their resistance. A year later, González-Guerrero *et al.* used this procedure to construct a planar fluxgate grown on a ferrite substrate [15]. The excitation coil was produced as described above, the detecting coils were made by evaporating and patterning Ti on glass and the ferromagnetic core consisted of an amorphous ferromagnetic stripe (Metglas 2714A). The use of a ferromagnetic substrate (ferrite) duplicates the exciting field without increasing the power consumption and also allows compensating superimposed magnetic fields up to tens of μT, by applying magnetic field pulses before measuring. The main drawback of the sensor is the presence of hysteresis in the response, slightly lower than 1 μT for a linear range of 80μT.

As mentioned above, there are two approaches for the miniaturization of the coils needed for making a fluxgate. At the beginning of the 21th century, the research in fluxgate sensors at GDM moves slowly to the second approach: miniaturization of the coils surrounding the ferromagnetic core. In the late 90's, the group of Prof. Martin Gijs at Ecole Polytechnique Fédérale de Lausanne proposed the use of Printed Circuit Board (PCB) Technology for the planarization and integration of fluxgate sensors [16,17]. The windings were made in PCB boards, the ferromagnetic core sandwiched between the boards and, in the last step, the boards are contacted to make a coil surrounding the core. In this way, ring-core fluxgates can be easily made, using a technology that is easy to integrate in the electronics industry.

The use of PCB technology offers an additional advantage to the efforts at GDM: the core could be directly electrodeposited in the PCB board, avoiding the use of glue to fix the magnetic amorphous core of the device. Researchers from Complutense University had been done extensive work in the electrodeposition of soft magnetic materials, in particular Co-P alloys, since the 80s.These Co-P electrodeposited alloys present a perpendicular anisotropy for thickness above 4μm [18], that increases the in-plane permeability and prevents their use in cores of the magnetic devices. Anisotropy can be controlled by modulating the composition in the perpendicular direction. Riveiro *et al.* reported amorphous Co-P multilayers consisting of alternating magnetic and non-magnetic layers with the easy axis in the plane, high permeability and a low coercivity (100 A/m) [19]. Coercivity can be reduced up to 3–10 A/m by modulating the composition using ferromagnetic layers with different P content [20].

**Figure 2.**
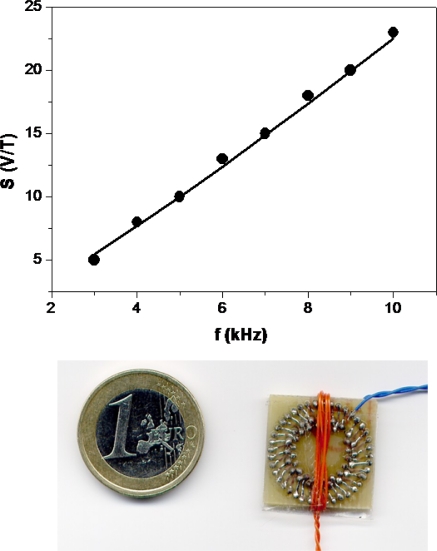
Sensitivity *vs*. frequency of former planar fluxgate developed by GDM’s group. Picture of a former version.

In 2004, Perez *et al.* reported a fluxgate sensor based on PCB technology, using a Co-P electrodeposited amorphous alloy as ferromagnetic core [21]. The use of Co-P as core increases the sensitivity compared to the sensors developed in Lausanne. The sensor shows a good linear behaviour up to 250 μT, without showing hysteresis, and a maximum sensitivity of 160 V/T at 50 kHz. They also developed an analytical model which accurately describes the dependence of the sensitivity on the frequency (Figure 2) and the amplitude of the driving current, two of the most important working parameters of this kind of sensors [22].

Recent work has been done on the dependence of the operation on the sensor as a function of the multilayered Co-P alloy used in the core. Although previous works suggested that a large gradient in composition in the perpendicular direction is needed to achieve good soft magnetic properties in CoP, Lucas *et al.* reported in 2006 that coercivity at high frequency is only due to eddy currents and, therefore, irrespective of the gradient in composition in the Co-P multilayered alloy [23]. Several sensors with different (Co_80_P_20_/Co_90_P_10_)_N_ cores have been made and characterized (where N is the number of bilayers in the multilayer). It has been shown that sensitivity does not depend on N, providing that N is high enough to have the easy axis in the plane, but the noise increases linearly with N, finding the best parameters for the Co-P multilayers for using them as magnetic core of fluxgate planar sensors [24].

In order to illustrate the progress in the miniaturization of fluxgates by GDM during the last two decades, we compare two different fluxgates. These are the one by Vincueria *et al.* [12] dating from 1994 and that one by Perez *et al.* [21] dating from 2004. See Table 1.

The first planar fluxgate by Vincueria had a sensitivity of 500 V/T (at 1 kHz and 8 mA driving current) and its area was 25 mm × 50 mm. It used some planar coils produced by lithography and sputtering techniques. The fluxgate by Perez *et al.*, has a size of 20 mm × 20 mm, translating into a reduction by a factor of 3 in the total area. This reduction leads to a decrease in the sensitivity to 160 V/T. However, the proportionality constant between sensitivity and size is exactly the same in both cases. This implies that any problem with the winding due to the miniaturization of the device has been completely overcome. As previously stated, this was achieved by micromachining the winding using PCB technology like done by Prof. Gijs [16,17].

Another important difference between both fluxgates is the nature of the magnetic core. While Vincueria *et al.* uses as magnetic core with a commercial Permalloy amorphous ribbon, that one used in the fluxgate by Perez *et al.* was produced by electrodeposition. Most of the research at GDM in electrodeposited materials has been addressed to the improvement of the soft magnetic properties of the core.

The main efforts in the miniaturization of the fluxgates have been considered: (i) reduction of the size while maintaining enough sensitivity for common applications like those described in section 3 and avoiding problems with the windings; and (ii) improving the procedure for their industrial fabrication. Much more research must be performed to improve the noise of the sensor. This requires consideration of the magnetic material used in the magnetic core since electrodeposited cores usually exhibit higher noise than bulk cores. The research to improve this important parameter was recently begun focussing on the spacecraft applications discussed in section 3.2 [24].

## 3. Applications

### 3.1. Signal Processing and Complex Systems with Planar Fluxgates

Fluxgates are commonly used for measuring DC and low frequency AC magnetic fields. The development of the double in-phase demodulation technique by GDM allowed not only to detect low intensity magnetic fields but to analyze their spectrum by using fluxgates [13].

Typically, fluxgates work on second harmonic principle and close-loop configuration. A lock-in amplifier (phase sensitive detector and amplifier) is used to obtain the second harmonic of the signal induced in the pickup or measuring coil of the fluxgate. The amplitude of the second harmonic in the pickup coil is proportional to the magnetic field to be measured. In conventional fluxgates the output of the lock-in is carried to a low pass filter to avoid high frequency noise, and the output is used as a feedback. This feedback improves the linearity of the response and increases the measurement range.

The GDM double demodulation technique is based on the use of two lock-in amplifiers or phase sensitive detectors synchronized with the same clock and the use of some frequency dividers and phase shifters. In this technique, the low pass filter and the feedback are suppressed to avoid a reduction in the high frequency content of the signal. Due to this open-loop configuration, the output of the first lock-in is modulated by any AC magnetic field to be measured. A second lock-in amplifier is used to demodulate this signal. This procedure can estimate the spectrum of any system excited with a magnetic field synchronized with the clock, or just the spectrum of any external magnetic field.

Fluxgates with the double in-phase demodulation have been produced by the GDM for different applications like tracking airplanes, radio frequency identification systems (RFID), *etc.* Planar fluxgates have been particular useful in the development of a new RFID system able to work not only in non-cleaned metal environments but through metals [25,26].

#### 3.1.1. Aircraft automatic identification and guidance

In the early 90s there was a worldwide trend to improve safety on ground operations in airports. In 1992, the Spanish Government decided to make a substantial R&D effort to prevent runway incursions and facilitate automatic guidance of aircraft along the airport movement areas. The aim was to improve the control systems and the guidance in surface movement of the aircrafts apart from preventing the runway incursions of non-authorized vehicles [27]. It was done through the RUSTEM project. One of the scopes of this project was to develop a system to perform in-land automatic detection and identification of airplanes and vehicles, and to improve their guidance. GDM developed a three axis fluxgate magnetometer within RUSTEM Project. The electronic and sensor head was especially design to support some extreme conditions of the airport like temperature, humidity, *etc.* The double in-phase demodulation was used. The excitation signal, filtering stages clocks, phase adjust and phase detections were derived from a unique master clock to avoid temperature effects. A pair of cables provided the path to command and control the fluxgates (auto-zero, reset, auto-calibration). The same cables were use for remote power feeding functions. The system allowed the compensation of external fields up to 300 μT, being 10 μT the dynamic range and 1nT the total noise. It was good enough to detect airplanes at 20 m of distance when placed in the taxi way axis or border. The device, which achieved the pursued objectives of airplane identification, quantification of aircraft speed and detection of non-authorized vehicles by means of the magnetic signature, ended with three patents related with the development [28–30].

#### 3.1.2. Planar fluxgates in RFID systems

The basic function of RFID systems is to send information from a mobile element without battery called tag to an antenna. The tag is fed by an external electromagnetic wave. The different available systems can be classified by the electromagnetic band used: (i) low frequency (LF: 125–134.5 kHz); (ii) High frequency (HF: 13.56 MHz); (iii) Ultra-high frequency (UHF∼900 MHz); and iv) Industrial, Scientific and Medical free band (ISM: 2.4 GHz). All of them show problems when metals are in the surrounding of the tag. It is due to screening or detuning effects coming from parasitic inductances produced by the metals and the eddy currents induced in these materials. All these problems can be avoided by reducing the work frequency. However, LF systems work through inductive couplings that become rapidly inefficient when reducing this parameter. A complete discussion of RFID in environments with metals can be found in [31].

GDM has developed a new RFID system [32] able to work at ultra-low frequencies (1–100 kHz). It is based on measuring the change of the magnetization of a magnetic material integrated in the tag. The system is composed of an antenna to produce a low frequency magnetic field, the tag and a reader.

The tag has a soft magnetic core with a winding around it. The AC magnetic field applied induces an electromotive force (e.m.f.) in the winding. The e.m.f. is used to feed a microcontroller. This one can short-circuit and open the winding. When the winding is short-circuited a current flows through it avoiding the magnetization of the magnetic core. The change of the magnetization when opening and short-circuiting the winding allows the microcontroller to send information.

The reader is a fluxgate in which the double demodulation is implemented. The planar fluxgate developed by Perez *et al.* [21] has been used. In fact, this technique is simplified for the RFID system because it is not needed to analyse the entire spectrum of the magnetic field since all the frequencies can be fixed.

The fluxgate is excited at a frequency *f*. The first demodulation to obtain the second harmonic is performed at a frequency *2f*. The applied magnetic field has a frequency *f_m_* ≪ *f*. The second demodulation is done at the frequency of the magnetization of the tag *f_m_*. All these signals are obtained from the same frequency generator and some frequency dividers. After the second lock-in it is obtained a signal that depends on the magnetization of the tag. It allows to detect the information emitted by the microcontroller in the tag.

RFID systems can be found in lots of different applications like control access, identification, tracking, automatic payment, *etc.* The new RFID system based on fluxgates can be used when the data transfer rate is not a crucial parameter and metals can cause problems. It can work through metals like aluminium layers up to 0.2 mm of thickness [26].

### 3.2. Planar Fluxgates for Space Applications

During the Cold War, many American and Soviet satellites carried on-board fluxgate magnetometers starting with the servo-oriented fluxgate of the Sputnik 3. Many of these fluxgates, together with other magnetic sensors (search coils and scalar sensors), were widely used to map the Earth’s, the interplanetary and other planets magnetic fields.

In Spain the National Institute for Aerospace Technology (INTA) was founded in 1942. The objective was to promote the aerospace research and the space science, and to support other industrial areas. However, Spain lacked a global space strategy until the 60s, when the national commission of space research (COIE) is founded to start an aeronautic activity [33].

Engineering projects like the first satellite INTASAT (launched in 1974), the INTA 255 rocket and the subsequent MINISAT-01 one (launched in 1997) did not carry magnetometers on board. However, in parallel with these developments, in the late 90s INTA started a program of nanosatellites. These platforms were conceived as micro and nano-technologic experiments test-beds. Consistent with this objective, the magnetometers used in the two NANOSAT spacecrafts launched in 2004 and 2009 respectively, were both based on micro and nanoscience technology. On board NANOSAT-01 there were two magnetic sensors: the ACS magnetic sensor based on four one-axis Anisotropic MagnetoResistance (AMR) Commercial Off-The-Shelf (COTS) sensors and an experimental sensor based on the Faraday Effect of a nanocomposed material. NANOSAT-1B carried another AMR COTS sensor and a MagnetoImpedance (MI) COTS sensor.

This strategy has been passed on to the development picosatellite OPTOS (to be launched in 2010), which will carry a three-axes AMR COTS sensor and a Giant MagnetoResistance (GMR) sensor. Here, the traditional bulky fluxgate sensors are clearly apart from the strategy of the nano and picosatellite missions [34]. Fluxgates in purely magnetic missions are normally used together with another magnetic sensor (vector or scalar) in dual configuration. It is, located in the same boom but at different distances (in the order of several meters) of the spacecraft. These magnetometers cover the Earth magnetic field (±65,000 nT) and achieve accuracies of 0.5 nT (equivalent to 3 arcsec approx.), and power consumptions in the order of 60 mW [10,35]. However, these magnetometers have two drawbacks facing applications in small satellites: they have masses (head and front-end) on the order of 300 g, which can mean the 1.5 % of the weight in a 20 kg platform like INTA nanosatellites, and they are expensive.

Miniaturized compact fluxgates with masses, lower than 100 g, prices in the order of 5,000 € (taking into account the tests for extreme conditions), and even wider dynamic range, but with poorer accuracies (on the order of nT) and noise in the order of 1 nT√Hz @ 1 Hz, are good candidates for technologic experimentation platforms. This is a driving reason for INTA, which in the last four years has made an effort to develop planar ring-core fluxgates integrated in a Printed Circuit Board (PCB).

**Figure 3.**
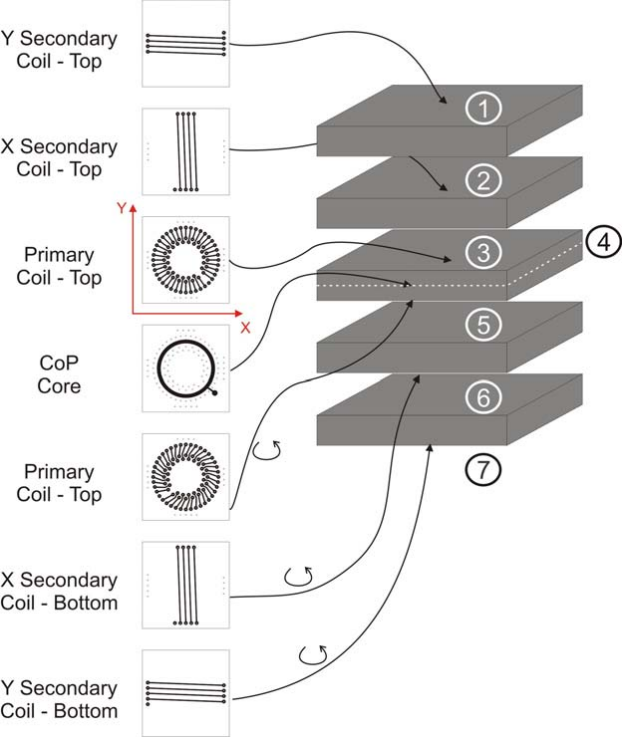
Scheme of the planar PCB-based fluxgate of INTA.

The magnetic head of actual prototypes under study are 1 cm^2^ surface sensors with 1.5 mm thickness. They are based in planar sensors developed in GDM [21] with a CoP electrodeposited core. The novelty is that both the primary and secondary coils are integrated in the different layers of the PCB (Figure 3) and that a multilayer core has been considered [20].

As it can be seen in Figure 3 the devices are two-axes magnetic sensors. The sensors are driven by an ac current at 50 kHz. Several studies have demonstrated that multilayer cores fluxgates have higher sensitivities 120 V/T) than bilayer ones (10 V/T). However for more than one bilayer, the sensitivity does not improve with the number of bilayers used. In contrast, the noise increases smoothly with the number of multilayers due to the pinning of the domain walls in the interfaces [24]. Also using multilayers the linear range is reduced up to values of ± 250 μT, which is still enough to cover the in-orbit Earth magnetic field and even some moderate to high fields expected in the anomalies of Mars. This sensor is being tested under extreme conditions. A positive result for the upscreening process would make it a good candidate for an experimental payload of a future small satellite of INTA.

Recently some Spanish groups have been involved in developing the magnetometers for the Laser Interferometer Space Antenna (LISA) Pathfinder mission. This mission is a precursor of a future LISA mission, which intends to demonstrate the technological capability to detect and observe gravitational waves from astronomical sources such as massive black holes and galactic binaries. One of the technological problems of this mission is to discern between magnetic and gravitational waves, and thus a very clean magnetic environment needs to be achieved (DC magnetic fields lower than 10 μT and fluctuations lower than 650 nT/√Hz, and magnetic field gradients lower than 5 μT/m with fluctuations lower than 25 nT/m/√Hz). Though the present magnetic sensing solution is still under consideration, of the current candidate magnetometers for LISA Pathfinder consist of four fluxgates magnetometers in the outer walls of a cavity to measure the magnetic field and gradient inside the cavity. In this mission the magnetometers will be space qualified magnetometers developed by Billingsey with a dynamic range of ± 100 μT and low noise up to 7 pT/√Hz @ 1 Hz, 200 g weight and 560 mW power consumption.

## 4. Conclusions and Future Trends

The research on fluxgate magnetometers started relatively late in Spain. Few groups have devoted their research to the improvement of these sensors. However, the study of planar fluxgates, one of the most trendy research lines in their miniaturization, began here.

The research carried out in the Group of Magnetic Devices (GDM) from the Complutense (UCM) and the Polytechnic University of Madrid (UPM) has allowed the development and fabrication of different fluxgates including planar-type in Spain. GDM also developed a signal processing technique to analyze the magnetic spectrum based on the use of fluxgates. Planar fluxgates with the double demodulation technique from GDM have demonstrated to be powerful instruments to be used in complex systems implying low frequency modulated magnetic fields. These devices will probably be applied to other security, recognition, non-destructive testing, and communication systems in a short and medium term.

To finish, in the National Institute of Aerospace Technology (INTA) strategy of miniaturized payloads for space applications, some effort has been made to achieve a compact fluxgate microsensor fully based on PCB technology for a future nano or picosatellite.

## Figures and Tables

**Table 1.** Comparison between two different fluxgates by GMD.

	**Vincueria *et al.* [12]**	**Perez *et al.*[21]**

**Year**	1994	2004

**Area**	25 mm × 50 mm	20 mm × 20 mm
**Sensitivity**	500 V/T	160 V/T
**Core**	Commercial amorphous ribbon	Electrodeposited soft magnetic multilayer
**Winding**	Planar	Micromachined
**Technologies**	UV-lithography, sputtering	UV-lithography, eletrodeposition, PCB
